# The Dynamic Changes of COL11A1 Expression During the Carcinogenesis and Development of Breast Cancer and as a Candidate Diagnostic and Prognostic Marker

**DOI:** 10.1155/tbj/7861864

**Published:** 2025-01-10

**Authors:** Yuli Wang, Jing Wang

**Affiliations:** ^1^Medical Laboratory, Tianjin Medical University General Hospital, Tianjin 300052, China; ^2^Tianjin Key Laboratory of Lung Cancer Metastasis and Tumor Microenvironment, Tianjin Lung Cancer Institute, Tianjin Medical University General Hospital, Tianjin 300052, China

**Keywords:** breast cancer, COL11A1, diagnosis and prognostic, dynamic changes

## Abstract

**Purpose:** Collagen type XI alpha 1 (COL11A1), a critical member of the collagen superfamily, is essential for tissue structure and integrity. This study aimed to validate previously identified variations in COL11A1 expression during breast cancer carcinogenesis and progression, as well as elucidate their clinical implications.

**Methods:** COL11A1 mRNA expression levels were assessed using real-time reverse transcription-PCR (RT-PCR) in 30 pairs of normal breast tissue and primary breast cancer, 30 pairs of primary breast cancer and lymph node metastases, 30 benign tumors, and 107 primary breast cancers. COL11A1 protein expression was evaluated by Western blot in six matched trios of normal tissue, primary cancer, and lymph node metastasis.

**Results:** COL11A1 mRNA levels were significantly higher in primary breast cancer tissues (*n* = 30) than in adjacent normal breast tissues (*p* < 0.001). Conversely, lymph node metastases (*n* = 30) showed significantly lower COL11A1 mRNA levels compared to their primary breast cancer counterparts (*p*=0.005). In a larger cohort, primary breast cancers (*n* = 107) had significantly elevated COL11A1 mRNA levels relative to adjacent normal tissues (*n* = 30) and benign tumors (*n* = 30) (*p* < 0.001). Benign tumors also demonstrated higher levels compared to normal tissues (*p*=0.012). The protein expression patterns were consistent with the mRNA findings. Receiver operating characteristic (ROC) curve analysis confirmed the diagnostic relevance of COL11A1 expression levels. Significant associations were found between COL11A1 mRNA levels and clinical parameters including lymph node involvement (*p*=0.046), clinical stage (*p*=0.004), and progesterone receptor status (*p*=0.048). Overexpression of COL11A1 was correlated with poor prognosis.

**Conclusions:** COL11A1 expression varies during breast tumor initiation and progression, with elevated levels linked to worse prognoses. These findings underscore COL11A1's potential as a biomarker in breast cancer, suggesting its assessment could enhance diagnostic and prognostic strategies for more personalized patient management.

## 1. Introduction

Collagens, the primary constituents of the interstitial extracellular matrix, are pivotal in orchestrating a wide array of biological functions. These functions span tissue development and morphogenesis, as well as cell differentiation and proliferation. Frequently, collagens are the matrix components most notably altered in solid tumors relative to healthy tissues. These alterations manifest in various forms, including changes in deposition, degradation, post-translational modifications, and organization [1]. Thus, the collagen-rich tissue stroma emerges as a critical focal point in cancer biology research, particularly due to its involvement in the malignant behaviors of tumors [2, 3]. Empirical evidence supports the significant pathophysiological roles collagens play across different cancer types [4, 5]. GEPIA2 database analysis revealed significant overexpression of five collagen types in breast cancer tissues: COL10A1, COL11A1, collagen type I alpha 1, collagen type XXII alpha 1, and collagen type III alpha 1, and they influence key processes like proliferation, metastasis, apoptosis, and drug resistance, shaping cancer progression and prognosis [6]. Collagen XVII may play a particularly important role in the growth and invasiveness of epithelial cancers [7]. For instance, collagen XVII has been highlighted for its crucial role in the progression and invasiveness of epithelial cancers. Moreover, collagen XII's upregulation in human breast cancer correlates with a dismal prognosis, suggesting its utility as a marker for identifying breast cancer patients at increased risk of metastatic relapse [8]. Additionally, elevated levels of collagen IV in metastatic breast cancer patients, as determined through immunohistochemistry, starkly contrast with those in healthy controls and primary breast cancer patients, underscoring the profound impact of collagen dynamics on cancer pathology [9].

COL11A1 is a crucial element of the ECM across a wide spectrum of cancers, with numerous reports highlighting its involvement in malignant tumor behavior [10]. Extensive research indicates that COL11A1 expression is elevated in various cancer types [11–13], including ovarian cancer, breast cancer [14–16], oral squamous cell carcinoma [17], lung adenocarcinoma [18, 19], and colorectal carcinoma [20], among others. A significant body of evidence [14], including data from the TCGA database, has documented the upregulation of COL11A1 in a range of human malignancies, such as adrenal cortical carcinoma, urothelial bladder carcinoma, invasive breast cancer, cervical squamous cell carcinoma and adenocarcinoma, cholangiocarcinoma, and colon adenocarcinoma. Notably, COL11A1 expression levels in breast cancer tissues surpass those in normal breast tissues, a finding corroborated by protein level assessments in breast cancer tissues versus normal breast tissues from the CPTAC database. Moreover, analyses of 20 pairs of breast cancer patient samples revealed that both mRNA transcription and protein expression levels of COL11A1 are significantly elevated in cancer tissues compared to adjacent nontumorous tissues. Similarly, COL11A1 levels are notably higher in colorectal cancer tissues than in normal tissues [21]. The association of COL11A1 expression with poor prognosis positions it as a promising candidate for therapeutic targeting [22]. Furthermore, Type XI collagen plays a pivotal role in the biology of pancreatic ductal adenocarcinoma and emerges as a potential noninvasive prognostic biomarker for patients with this condition [23]. Our preliminary findings, awaiting publication, indicate an upregulation of COL11A1 in primary breast cancer relative to adjacent normal tissue, while microarray analysis reveals its downregulation in lymph node metastasis compared to primary breast cancer, underscoring the complex role of COL11A1 in cancer dynamics [24].

Epidemiological studies have highlighted the pivotal role of cancer prevention in diminishing global mortality rates [25]. Breast cancer, which ranks as the most prevalent cancer among women, stands as the foremost cause of cancer-related deaths globally among this demographic [26]. Consequently, delving into the molecular mechanisms and identifying diagnostic and prognostic markers critical for the onset and progression of breast cancer is of paramount importance. In this study, we investigate the role of COL11A1 in breast cancer development and assess its clinical relevance in diagnosing and prognosticating the disease. We quantified COL11A1 mRNA levels in 30 pairs of adjacent normal breast and primary breast cancer tissues, 30 pairs of primary breast cancer and lymph node metastasis tissues, 30 benign tumor tissues, and 107 primary breast cancer tissues using real-time reverse transcription (RT)-PCR. This analysis aims to elucidate the relationship between COL11A1 mRNA levels and the progression of breast cancer. Furthermore, we examine COL11A1 protein expression in six sets of matched samples encompassing adjacent normal breast, primary breast cancer, and lymph node metastasis tissues. Our findings indicate that the dynamic alterations in COL11A1 gene expression are intricately involved in both the carcinogenesis and development of breast cancer, underlining the potential of COL11A1 as a significant diagnostic and prognostic marker.

## 2. Materials and Methods

### 2.1. Patients and Samples

Between January 2002 and October 2003, tissue samples from 107 breast cancer patients were collected post-surgery at Tianjin Cancer Hospital, China. These samples were immediately snap-frozen in liquid nitrogen and stored at −80°C to preserve their integrity. Histopathological assessments were rigorously performed on all specimens post-surgical resection to confirm diagnostic details. For the purposes of real-time RT-PCR and Western blot analyses, only those samples containing 75% or more tumor cells were included in the study. Specifically, for the real-time RT-PCR component, primary breast cancer tissues and matched adjacent normal breast tissues were collected from 30 cases, and primary breast cancer tissues along with matched lymph node metastasis tissues were collected from another 30 cases. Among these, 10 sets of matched samples comprising adjacent normal breast tissue, primary breast cancer tissue, and lymph node metastasis tissue from the same patient were identified, with 6 sets subsequently used for Western blot analysis. The patient demographics, encompassing age, tumor size, nodal status, number of involved lymph nodes, clinical stage, histological grade, and the status of estrogen receptor (ER), progesterone receptor (PR), and Her-2, are detailed in Table 1. Additionally, 30 benign tumor specimens, including 27 fibroadenomas and 3 cases of fibrocystic disease, were also collected following surgery at the same institution. The collection and use of these tissue samples received the approval of the Institutional Review Board and the Research Ethics Committee, ensuring adherence to ethical guidelines.

### 2.2. RNA Extraction

Total RNA was extracted from clinical specimens utilizing Trizol reagent (Invitrogen, Gaithersburg, MD, USA), strictly adhering to the protocol provided by the manufacturer. The concentration and purity of the RNA were quantified through spectrophotometric analysis, measuring absorbance at 260 nm and 280 nm wavelengths. To evaluate the integrity of the RNA, formaldehyde agarose gel electrophoresis was employed. Upon extraction, the RNA was preserved at −80°C for future analyses.

### 2.3. RT

Total RNA served as the template for the RT reaction, which was conducted in a 20 μL volume using the SUPERSCRIPT First-Strand Synthesis System (Invitrogen) following the manufacturer's guidelines. In summary, 5 μg of total RNA was mixed with 0.5 μg of oligo (dT), 10 mmol of a dNTP mix, and diethylpyrocarbonate-treated double distilled H2O (DEPC-ddH2O). This mixture was initially heated for 5 min at 65°C and then rapidly cooled on ice. Subsequently, the mix was incubated for 50 min at 42°C upon addition of 4 *μ*L First-Strand Buffer, 0.2 *μ*mol DTT, 40 units of RNaseOUT ribonuclease inhibitor, and 200 units of SuperScript II enzyme. The reaction was terminated by heating at 70°C for 15 min. The resulting cDNA was preserved at −20°C for future applications.

### 2.4. Real-Time RT-PCR Assay

Primers and a TaqMan probe for COL11A1 were developed using Oligo 6.0, with sequences: forward primer 5′-TCGCATTGACCTTCCTCTTC-3′, reverse primer 5′-TCCCGTTGTTTTGATATTC-3′, and probe 5′-CAGAGGAGCTGCTCCAGTTGATGTA-3′. These were BLASTed against NCBI GenBank for verification [27]. We quantified transcripts of the housekeeping gene glyceraldehydes 3-phosphate dehydrogenase (GAPDH) as control, as described [28]. Forward primer, 5′-GAAGGTGAAGGTCGGAGTC-3′; reverse primer, 5′-GAAGATGGTGATGGGATTTC-3'; and probe, 5′-CAAGCTTCCCGTTCTCAGCC-3′. The TaqMan probe was labeled with FAM (6-carboxy-fluorescein) at the 5′ end and TAMRA (6-carboxy-tetramethyl-rhodamine) at the 3′ end.

Real-time PCR utilized the ABI Prism 7500 with fluorescent TaqMan probes for continuous monitoring. Amplification used the Platinum Quantitative RT-PCR ThermoScript One-Step System in 20 μL reactions with 40 ng of cDNA, 1 μM primers, 0.2 μM probe, and 10 μL SuperMix-UDG, following: 95°C for 3 min, then 40 cycles of 95°C for 30 s and 60°C for 1 min. Fluorescence was measured at each annealing phase, with results and plots analyzed automatically post-run. The mRNA expression levels were relatively quantified by normalizing the cycle threshold (CT) value of COL11A1 against the CT value of GAPDH from the identical RNA sample. This normalization was achieved by calculating the difference in CT values (△CT = CT_COL11A1_ ^−^ CT_GAPDH_). The relative expression level of COL11A1 was then determined using the formula 2^−△CT^, offering a precise measure of gene expression.

### 2.5. Western Blot Analysis

Total cellular protein was isolated from whole tissue samples through homogenization in a lysis buffer composed of 20 mM Tris-HCl (pH 7.4), 150 mM sodium chloride, 5 mM EDTA, and 1% Triton X-100. Following homogenization, cell lysates were subjected to centrifugation at 12,000 g for 15 min at 4°C, after which the insoluble debris was removed. The protein concentration in the supernatant, now clarified cell lysate, was determined using a protein assay reagent (Bio-Rad, Hercules, CA, USA) within 96-well plates, employing BSA as the calibration standard. This resultant total cellular lysate was then either directly used for SDS-PAGE analysis or stored at −80°C for future experiments.

For the Western blot analysis, cell extracts (40 *μ*g) were prepared in loading buffer, consisting of 500 mmol/L Tris-HCl (pH 6.8), 20% mercaptoethanol, 10% glycerol, 10% SDS, and 0.05% bromophenol blue. The samples were heated for 2 min at 95°C before being run on 10% SDS-polyacrylamide gels (Invitrogen, Carlsbad, CA, USA). A lane in each gel was reserved for the BenchMark Pre-Stained Protein Ladder (Invitrogen, USA) to facilitate protein size identification. Following electrophoresis, proteins were transferred onto Immun-Blot polyvinylidene difluoride membranes (Bio-Rad Laboratories, CA, USA). The membranes were then blocked using 5% nonfat dry milk in Tris-buffered saline (TBS, 20 mM Tris, 137 mM NaCl, pH 7.5) with 0.1% Triton X-100 for 1 h at room temperature. Antibodies specific to COL11A1 (rabbit-anti-human, Calbiochem, CA, USA) and *β*-actin (goat-anti-human, Santa Cruz, CA, USA) were applied as per the manufacturer's instructions. The primary antibodies were incubated with the membranes overnight at 4°C with gentle agitation. This was followed by the application of corresponding horseradish peroxidase–conjugated anti-rabbit and anti-goat secondary antibodies (Santa Cruz, CA, USA) at a 1:1000 dilution. Secondary antibody incubation was conducted at room temperature for 45 min. Detection was achieved using enhanced chemiluminescence (ECL; Amersham Biosciences, Piscataway, NJ) and visualized on medical X-ray film (Konica Minolta, Japan). Image capture and quantification were performed using a Gel Doc XR System and Quantity One software (Bio-Rad, Hercules, CA, USA), respectively, facilitating the analysis of protein expression levels.

### 2.6. Statistical Analyses

Paired *t*-test, Student's *t*-test, and one-way ANOVA were used where appropriate to assess clinical variables as well as COL11A1 mRNA expression levels. Receiver operating characteristic (ROC) was constructed to investigate the sensitivity and specificity. Survival rates were estimated by the Kaplan–Meier method. All statistical analyses as described above were performed using SPSS13.0 software. *p* values less than 0.05 were considered to be significant.

## 3. Results

### 3.1. COL11A1 Expression Profiles in Primary Breast Cancer: Comparison With Normal Tissues and Lymph Node Metastases

**Figure 1:**
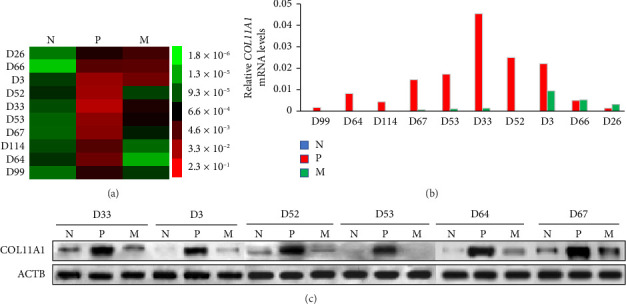
(a, b) Differential expression of COL11A1 mRNA in adjacent normal (N), primary (P), and lymph node (M) breast tumors. (c) Differential expression of COL11A1 protein in adjacent normal (N), primary (P), and lymph node (M) breast tumors. COL11A1 mRNA and protein expression levels were significantly higher in breast cancer specimens compared with matched normal breast specimens (*n* = 30, *p*=0.001). In contrast, in lymph node metastasis tissues, COL11A1 mRNA levels were significantly lower compared with matched primary carcinoma (*n* = 30, *p*=0.005).

In our analysis, COL11A1 mRNA expression levels were markedly elevated in breast cancer specimens relative to matched normal breast specimens (*n* = 30, *p* < 0.001). Conversely, within lymph node metastasis tissues, we observed a significant decrease in COL11A1 mRNA levels compared to their matched primary carcinoma tissues (*n* = 30, *p*=0.005). Notably, within the same cohort of patients, a rise in COL11A1 mRNA levels was documented from the matched normal breast to primary breast cancer, with a subsequent decline from the primary breast cancer to the matched lymph node metastasis tissues (*n* = 10). This pattern is visually represented in Figure 1. Similarly, the trend in COL11A1 protein expression mirrored the mRNA expression patterns (also depicted in Figure 1), providing a robust confirmation of the real-time RT-PCR findings.

### 3.2. COL11A1 mRNA Expression in Normal Breast, Benign Tumor, Primary Breast Cancer, and Lymph Node Metastases

**Figure 2:**
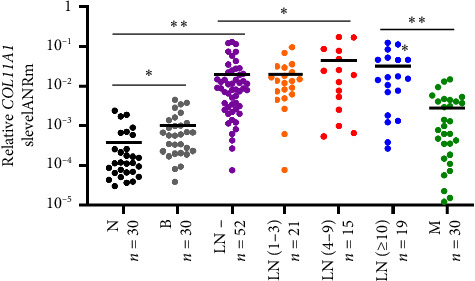
Dynamic changes of COL11A1 mRNA expression in adjacent normal breast tissues (N), benign tumors (B), lymph node-negative tissues (LN (0)), primary breast cancer tissues with one to three involved axillary lymph nodes (LN (1–3)), primary breast cancer tissues with four to nine involved axillary lymph nodes (LN (4–9)), primary breast cancer tissues with ten or more axillary lymph nodes (LN (≥ 10)), and lymph node metastasis tissues (M). Horizontal bar represents median in each group.

**Figure 3:**
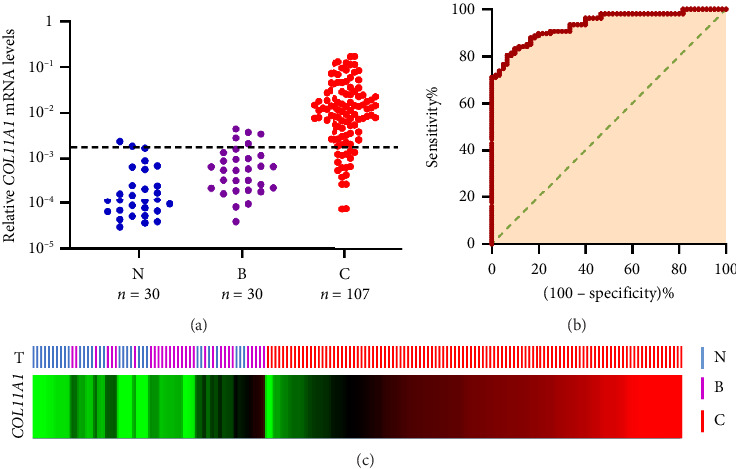
(a, c) COL11A1 mRNA expression in adjacent normal breast tissues (N), benign breast tissues (B), and primary breast cancer tissues (C). Horizontal bar represents cutoff point (2.0E − 03). (b) Receiver operating characteristic (ROC) curve analysis: area = 0.935, standard error = 0.018. The optimum cutoff level for the differentiation between adjacent normal breast, benign breast, and primary breast cancer was determined as the point that provides the greatest sum of sensitivity and specificity, in this case a level of > 2.0E − 03.

Average COL11A1 mRNA expression levels gradually increased from normal breast (n=30), benign tumors (*n* = 30), LN (0) tumors (*n* = 52), LN (1–3) tumors (*n* = 21) to LN (4–9) tumors (*n* = 15), and then decreased in LN (≥ 10) tumors (*n* = 19), as shown in Figure 2. Remarkably, the average COL11A1 mRNA expression in primary breast cancer samples (*n* = 107) was found to be 67.4 times greater than that in normal samples (*n* = 30, *p* < 0.001) and 25 times higher than in benign tumor samples (*n* = 30, *p* < 0.001), detailed in Figures 3(a) and 3(c). Additionally, benign tumor tissues displayed significantly higher COL11A1 mRNA levels compared to normal tissues (*p*=0.012). Through ROC curve analysis, an optimal cutoff point (2.0E − 03) was identified to differentiate between adjacent normal breast, benign tumor, and primary breast cancer tissues, as depicted in Figure 3(b). Notably, 96.9% (29/30) of adjacent normal breast tissues and 83.3% (25/30) of benign tumor tissues exhibited low COL11A1 mRNA expression levels, in contrast to 81.3% (87/107) of primary breast cancer samples which demonstrated high COL11A1 mRNA expression.

### 3.3. COL11A1 mRNA Expression in Relation to Clinical Factors

Table 1 illustrates the relationship between COL11A1 mRNA levels and various clinicopathological factors. Tumors classified as stage I to III exhibited significantly lower COL11A1 transcript levels compared to those at stage IV (*p*=0.004). Additionally, tumors from patients expressing the PR showed reduced COL11A1 mRNA levels relative to those lacking PR expression (*p*=0.048). Furthermore, tumors in patients with fewer metastases in the involved lymph nodes (0–3) presented lower COL11A1 transcript levels (*p*=0.046) than those in patients with a higher count of metastatic lymph nodes (4–9). While there was no significant correlation with patient age, tumor size, histological grade, Her-2 status, or ER status, an apparent trend was noted, indicating increased COL11A1 expression associated with larger tumor sizes and higher histological grades.

### 3.4. Prognostic Analysis of COL11A1 mRNA Expression in Breast Cancer Tissues

**Figure 4:**
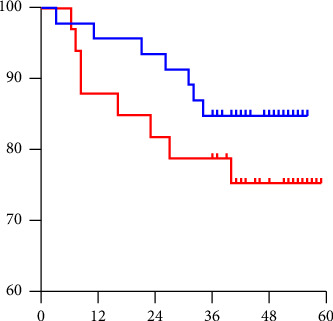
Survival analyses based on the mRNA level of COL11A1. Patients were divided into two groups: solid line, those with COL11A1 mRNA levels lower than 1.0E − 02; dotted line, those with levels of 1.0E − 02 or higher. Patients with low COL11A1 mRNA expression had a significantly better disease-free survival than those with high COL11A1 mRNA expression.

In the survival analysis, patients were categorized into two groups according to their COL11A1 mRNA levels: those with low levels (below 2.0E − 02, comprising 54 cases) and those with high levels (2.0E − 02 or above, comprising 29 cases). We compared the survival rates between these two groups. Over the course of a 3-year follow-up, a significant correlation was found between high COL11A1 levels and poor prognosis, as depicted in Figure 4.

## 4. Discussion

The association between COL11A1 and malignant tumors has only been investigated in a few published articles, including our previous study [10, 24, 29]. In this study, we found that both mRNA and protein levels of COL11A1 were elevated in primary breast cancer tissues compared to normal breast tissue, yet decreased in lymph node metastases relative to primary breast cancer. Significant associations were identified between COL11A1 mRNA levels and factors such as lymph node involvement, clinical stage, and PR status. However, no significant correlations were observed between COL11A1 mRNA expression and other clinical characteristics, including patient age, tumor size, histological grade, Her-2 status, and ER status. ROC analysis further validated the utility of COL11A1 as both a diagnostic and prognostic marker, reinforcing its potential significance in cancer diagnostics and patient prognosis assessment.

Our findings reveal that COL11A1 mRNA and protein expressions are heightened in primary breast tumors relative to their normal counterparts. This observation aligns with a study [20] analyzing the TCGA colorectal cancer dataset, which reported a notable increase in COL11A1 mRNA expression across various cancer subtypes, including colon adenocarcinoma, colon mucinous adenocarcinoma, rectal adenocarcinoma, and cecal adenocarcinoma, when compared to normal colon tissues. Furthermore, recent investigations employing microarray technologies have consistently identified COL11A1 overexpression in several cancer types (such as breast cancer, lung adenocarcinoma, and hypopharyngeal cancer) versus their corresponding normal tissues [30–32]. These studies underscore a widespread pattern of COL11A1 upregulation across a diverse array of malignancies, encompassing adrenal cortical carcinoma, urothelial bladder carcinoma, invasive breast cancer, cervical squamous and adenocarcinoma, cholangiocarcinoma, and colon adenocarcinoma [14]. In line with these findings, our research indicates that COL11A1 mRNA levels are capable of distinguishing between normal breast tissue, benign tumors, and cancerous tissues, hinting at its utility as an adjunct molecular marker for breast cancer diagnosis. Collectively, these results underscore the pivotal role of COL11A1 in the progression of mammary tumorigenesis.

We noted that COL11A1 mRNA levels are elevated in breast cancer tissues relative to adjacent normal and benign tissues. It has been reported that COL11A1 may be a potential prognostic marker for colorectal cancer [33]. COL11A1 overexpression significantly correlated with pathological stage in non-small-cell lung cancer [34]. The study revealed through gene expression profiling that COL11A1 expression escalates during ovarian cancer progression, serving as an indicator of poor clinical outcomes in patients with epithelial ovarian cancer [35]. Overexpression of COL11A1 has been associated with an adverse outcome in a variety of primary cancers, including breast [36] and pancreas [37]. Furthermore, our data compellingly indicate that elevated COL11A1 mRNA levels in the primary tumor correlate with a poorer prognosis. This correlation is validated by the Kaplan–Meier survival curve (Figure 4), illustrating that patients with higher COL11A1 levels experience increased instances of metastasis, recurrence, or death compared to those with lower COL11A1 expression in the primary tumor. High COL11A1 expression strongly associated with poor prognosis in breast cancer [14, 15]. Collectively, these findings establish a clear association between high COL11A1 expression and poor prognosis.

Additionally, we noted a reduction in COL11A1 expression levels in lymph node metastases relative to their corresponding primary breast tumors, a phenomenon that remains unexplained currently. The intricacies of tumor invasion, encompassing cell migration and interactions with the microenvironment at distant sites, contribute to the complexity of this observation. Metastasis stands as one of the most perplexing aspects of cancer biology, representing a complex, sequential, and interconnected process that culminates in the development of distant secondary tumors. This series of events is known collectively as the invasion–metastasis cascade [38]. To validate the credibility of this study, we demonstrated through real-time RT-PCR that COL11A1 mRNA expression levels are significantly elevated in primary breast cancer tissues compared to lymph node metastasis tissues. The pattern of dynamic gene expression changes throughout cancer progression has been established for numerous genes [39]. The fluctuating expression of COL11A1 throughout the stages of carcinogenesis and breast cancer development implies a dual role for COL11A1 in tumor progression. On the one hand, the dynamic shifts in COL11A1 expression could be driving factors behind the advancement of breast cancer. On the other hand, these changes might result from the tumorigenic transformations within the cell, possibly occurring in tandem. The precise contribution of COL11A1 to tumor progression and metastasis remains undefined and could vary significantly depending on the cancer's stage of development. COL11A1 promotes invasion in cutaneous squamous cell carcinoma [10]. Metastasis is contingent upon the interplay between tumor cells and the host's cellular environment. While oncogenesis and tumor progression are interconnected, they represent distinct processes. Our findings suggest that COL11A1 might function as a metastasis suppressor in the advanced stages of breast cancer progression, highlighting its potential role in the complex dynamics of tumor spread.

Wu and colleagues [40] conducted a preliminary study on the structural and functional characteristics of the COL11A1 gene promoters, finding that COL11A1 features a TATA-less promoter, which, akin to other TATA-less promoters, possesses multiple transcription start sites. Moreover, the loss of expression of the COL4A5 and COL4A6 chains in colorectal cancer has been linked to the hypermethylation of their promoter regions [41]. This suggests that dynamic chromatin remodeling (e.g., hypermethylation) at the COL11A1 promoter could be a key factor influencing changes in COL11A1 expression. Consequently, the regulation of COL11A1 expression is likely subject to a variety of molecular mechanisms.

In conclusion, our findings clearly demonstrate a significant correlation between breast cancer progression and alterations in COL11A1 expression. The changes in COL11A1 mRNA levels emerge as a promising diagnostic and prognostic marker for breast cancer, effectively reflecting the progression of the disease. This insight underscores the potential of COL11A1 expression profiling to enhance early detection, improve prognostic accuracy, and inform personalized treatment strategies in breast cancer management.

## Figures and Tables

**Table 1:** Correlation between the mRNA levels of COL11A1 and clinicopathologic factors.

Clinicopathologic factors	Cases	($\bar{x}\pm \text{SD}$) × 10^−2^	95% CI (× 10^−2^)	*p*
Patients' age				
< 50	49	2.84 ± 3.63	1.77, 3.83	0.350
≥ 50	58	2.19 ± 3.56	1.26, 3.17	
Tumor size (cm)				
≤ 2	21	1.96 ± 2.24	9.46, 29.81	0.559
2–5	71	2.47 ± 3.56	1.63, 3.32	
> 5	15	3.28 ± 5.09	0.46, 6.10	
Lymph node status				
Negative	52	1.94 ± 3.01	1.10, 2.78	0.129
Positive	55	3.00 ± 4.02	1.91, 4.09	
Lymph node positive number				
0–3	73	1.94 ± 2.81	1.29, 2.60	0.046⁣^∗^
4–9	15	4.30 ± 5.76	1.10, 7.49	
≥ 10	19	3.14 ± 3.77	1.32, 4.96	
Clinical stage				
I	15	1.74 ± 1.72	0.78, 2.69	0.004⁣^∗∗^
II	66	2.14 ± 3.10	1.38, 2.90	
III	22	2.94 ± 4.57	0.91, 4.97	
IV	4	8.51 ± 5.63	−0.45, 17.43	
Histological grade				
I	12	1.61 ± 3.63	−0.69, 3.92	0.493
II	72	2.43 ± 3.34	1.65, 3.22	
III	23	3.11 ± 4.33	1.24, 4.99	
Estrogen receptor				
Positive	66	2.73 ± 3.84	1.80, 3.71	0.338
Negative	37	2.01 ± 3.29	0.93, 3.06	
Missing	4			
Progesterone receptor				
Positive	46	1.73 ± 2.25	1.07, 2.38	0.048
Negative	57	3.08 ± 4.41	1.91, 4.29	
Missing	4			
Her-2/neu expression				
Negative	60	2.47 ± 3.79	1.49, 3.45	0.930
Positive	33	2.54 ± 3.44	1.32, 3.83	
Missing	15			
Relapse or distant metastasis⁣^∗∗∗^				
Negative	64	20.0 ± 30.5	12.0, 27.0	0.164
Positive	19	36.0 ± 46.1	14.0, 58.0	
Missing	24			

⁣^∗^Primary tumors with 0–3 positive lymph nodes had significantly lower levels of COL11A1 mRNA compared with positive lymph node number 4–9 tumors.

⁣^∗∗^Stage I ∼ III tumors had significantly lower COL11A1 transcripts than stage IV tumors.

⁣^∗∗∗^Cases were followed up more than 3 years.

## Data Availability

The data that have been used are confidential.
